# Editorial: Paediatric Cardiomyopathies

**DOI:** 10.3389/fped.2021.696443

**Published:** 2021-05-11

**Authors:** Emanuele Monda, Juan Pablo Kaski, Giuseppe Limongelli

**Affiliations:** ^1^Inherited and Rare Cardiovascular Diseases, Department of Translational Medical Sciences, University of Campania “Luigi Vanvitelli”, Monaldi Hospital, Naples, Italy; ^2^Centre for Inherited Cardiovascular Diseases, Great Ormond Street Hospital, London, United Kingdom; ^3^Institute of Cardiovascular Sciences, University College London, London, United Kingdom

**Keywords:** cardiomyopathies, sudden cardiac death, dilated cardiomyopathy, LMNA cardiomyopathy, myocarditis

Paediatric cardiomyopathies (CMPs) are rare disorders associated with significant morbidity and mortality (1). They represent a varied group of diseases, with the outcome largely dependent on phenotype, aetiology, and clinical characteristics (2, 3). Data in the literature on paediatric CMPs are scant, leading to several “gaps in knowledge.” In this Research Topic, experts in the field explored different topics contributing to increase knowledge on this challenging area.

Sudden cardiac death (SCD) in paediatric CMPs is a rare but devastating event. Since SCD sometimes represents the first manifestation of the disease, the identification of the specific underlying disorder and the risk factors for SCD is crucial to prevent such event (4). However, evidence on risk stratification and preventing strategies is lacking. Furthermore, paediatric patients undergoing implantable cardioverter defibrillator (ICD) implantation show an elevated rate of short- and long-term device-related complications. In their review entitled “Sudden Cardiac Death in Children Affected by Cardiomyopathies: An Update on Risk Factors and Indications at Transvenous or Subcutaneous Implantable Defibrillators”, Rella et al. extensively discussed this topic providing an overview on risk factors for SCD and the possible indication for subcutaneous vs. transvenous ICD in patients with paediatric CMPs.

Although arrhythmic complications are well-described in children with hypertrophic cardiomyopathy (HCM), their importance in paediatric dilated cardiomyopathy (DCM) is less well-understood. In the article “Correlation Between Arrhythmia and the Prognosis in Children With EFE/LVNC/DCM”, Wang et al. investigated 31 children with endomyocardial fibrosis (EFE), left ventricular non-compaction (LVNC), or DCM with tachyarrhythmias [including supraventricular tachycardia (SVT), atrial fibrillation (AF), atrial flutter (Af), ventricular tachycardia (VT), frequent premature ventricular contraction (PVC), and Wolff–Parkinson–White syndrome B (WPW-B)] or interventricular blocks (IVB) or complete left bundle branch block (CLBBB) aiming to explore the correlation between different phenotypes of arrhythmias and prognosis in these patients. Forty-two children with EFE with or without arrhythmias were also included to assess the impact of arrhythmias on prognosis. The authors showed that children with EFE and WPW-B that manifested SVT, IVB, or CLBBB carry worse outcome in terms of time to recovery of left ventricular ejection fraction (LVEF) and left ventricular end-diastolic diameter (LVEDD), compared to children with EFE and AF/Af or VT. Moreover, children with EFE and arrhythmias had worse overall outcomes compared to those without. Thus, the authors concluded that the long-term outcome of children with CMPs is associated with the type of arrhythmia, suggesting that early arrhythmia control is required to improve the prognosis of these patients.

Myocarditis is an inflammatory cardiac disease that may occur as a consequence of infection, exposure to toxic substances, and immune systemic activation. It is a relatively common cause of SCD, DCM, and heart failure in children. The clinical presentation of myocarditis ranges from subclinical disease to fulminant heart failure, the latter representing a common presentation in young children. Viral infection is the main cause of myocarditis in children. In addition to direct viral infection, myocardial damage is partly mediated by immunological mechanisms both in the acute and chronic phases of the disease. Immunosuppressive therapy could therefore theoretically improve prognosis in selected cases (e.g., giant cell myocarditis, myocarditis associated with known extra-cardiac autoimmune disease) (5). In the study entitled “Immunosuppressive Treatment for Myocarditis in the Paediatric Population: A Meta-Analysis”, He et al. performed a systematic meta-analysis of studies investigating the efficacy of immunosuppressive treatment in the paediatric population with acute myocarditis. They found that short-term immunosuppressive treatment may improve LVEF, reduce LVEDD, and reduce the risk of death and heart transplantation in children with myocarditis. However, according to the authors, these results should be interpreted cautiously because of several study limitations, such as, among others, the small sample size, publication bias, lack of data on the viral genome and histologic type of myocarditis.

Mutations in the LMNA gene are responsible for a wide spectrum of diseases, known as laminopathies, including peripheral neuropathy, skeletal muscle disorders, and DCM. LMNA-related CMPs are frequently associated to SVT, conduction disorders, and less commonly to VT, potentially leading to SCD. Although cardiac involvement in laminopathies is well-described in adults, there are little data on laminopathies in children. In the study entitled “Cardiovascular Involvement in Paediatric Laminopathies. Report of Six Patients and Literature Revision”, Baban et al. described six children with LMNA mutations. Arrhythmic events and/or DCM represented the most common type of myocardial involvement, while neuromuscular involvement was mild or absent. Importantly, they observe the coexistence of congenital heart defects (CHDs) and aortic involvement in four patients, suggesting for the first time a possible causative role of LMNA variant in left-sided CHDs and progressive aortopathies. However, further studies are essential to confirm this observation.

The editors hope that readers of this Research Topic will find it of interest.

## Author Contributions

EM, JK, and GL contributed equally to the conception and drafting of the Editorial. All authors contributed to the article and approved the submitted version.

## Conflict of Interest

The authors declare that the research was conducted in the absence of any commercial or financial relationships that could be construed as a potential conflict of interest.
